# Multidisciplinary approach and clinical management of McCune-Albright syndrome

**DOI:** 10.3389/fendo.2026.1786683

**Published:** 2026-04-02

**Authors:** Jingna Wang, Yanmei Sang

**Affiliations:** 1 Baoding Hospital of Beijing Children’s Hospital, Capital Medical University, Baoding, Hebei, China; 2 Department of Pediatric Endocrinology, Genetic and Metabolism, National Center for Children’s Health, Beijing Children’s Hospital of Capital Medical University, Beijing, China

**Keywords:** clinical management, McCune-Albright syndrome (MAS), multidisciplinary approach, rare, sporadic mosaic disorder

## Abstract

McCune-Albright syndrome (MAS) is a rare, sporadic mosaic disorder classically characterized by fibrous dysplasia (FD) of bone, café-au-lait macules, and endocrine hyperfunction. The estimated prevalence ranges from 1 in 100,000 to 1 in 1,000,000 individuals, with no clear differences across ethnic groups. The central pathogenic mechanism involves postzygotic somatic gain-of-function mutations in the *GNAS* gene, which encodes the α subunit of the stimulatory G protein (Gsα). The currently identified pathogenic variants are concentrated at codons R201 (e.g., R201H and R201C) and Q227. This review systematically summarizes the molecular genetic basis, phenotypic heterogeneity, histopathological and imaging characteristics, diagnostic strategies, precision treatment approaches, and long-term prognosis of MAS. By integrating recent advances, we also highlight emerging research directions and provide a structured framework to support clinical diagnosis and multidisciplinary management.

## 1 Genetic pathogenesis

### 1.1 Genetic basis

MAS results from postzygotic somatic activating mutations of the *GNAS* gene located on chromosome 20q13.32 (1–3). Approximately 95% of pathogenic variants occur in exon 8, leading to substitution of arginine at codon 201 with histidine (NM_000516.5; c.602G>A, p.Arg201His) or cysteine (NM_000516.5; c.601C>T, p.Arg201Cys). The remaining ~5% of mutations are found in exon 9 and result in substitution of glutamine at codon 227 with leucine (NM_000516.4; c.680A>T, p.Glu227Leu), a variant more frequently reported in isolated FD than in classical MAS (4, 5). To date, all *GNAS* variants associated with MAS are missense mutations, and no insertions, deletions, or truncating mutations have been identified. Zhadina et al. (6) reported that the R201H mutation occurs more frequently than R201C overall. Moreover, R201C is more prevalent among Black/African American and White populations, whereas R201H is relatively more prevalent in Asian populations. Notably, all reported *GNAS* mutations in MAS are *de novo*, and no confirmed cases of vertical transmission have been reported.

Functionally, these activating mutations result in constitutive activation of Gsα, leading to persistent stimulation of adenylyl cyclase (AC) and chronically elevated intracellular cyclic adenosine monophosphate (cAMP) levels. Sustained cAMP signaling dysregulates downstream transcriptional and metabolic pathways, producing functional abnormalities across multiple target tissues, including bone, skin, and endocrine organs. Because these mutations arise during embryogenesis, affected individuals exhibit somatic mosaicism. The earlier the mutations occur, the more germ layers and tissues are affected. Conversely, mutations occurring later in embryonic development lead to more restricted organ involvement and incomplete clinical phenotypes. Consequently, the timing, distribution, and proportion of mutant cells largely determine the clinical phenotypic heterogeneity observed in MAS (7).

### 1.2 Abnormal signaling pathways

The multisystem manifestations of MAS reflect tissue-specific consequences of aberrant cAMP signaling. In the skeletal system, chronically elevated cAMP levels promote differentiation of mesenchymal stem cells toward a fibroblastic lineage while impairing normal osteoblast maturation and bone remodeling, ultimately resulting in FD (8). In endocrine tissues, activating *GNAS* mutations cause ligand-independent activation of hormone receptors, including those for thyroid-stimulating hormone, follicle-stimulating hormone, and adrenocorticotropic hormone (ACTH), leading to autonomous hormone secretion and clinical features such as precocious puberty and hyperthyroidism (9). In the skin, increased cAMP signaling in melanocytes enhances tyrosinase activity and melanin synthesis, producing the characteristic café-au-lait macules (3).

## 2 Age of onset and clinical features

The clinical manifestations of MAS are strongly age dependent, with distinct patterns emerging across different stages of life. Cutaneous lesions may be apparent in the neonatal period, endocrine abnormalities often manifest during early childhood, and skeletal complications may progress throughout adolescence and adulthood. In addition, patients remain at risk for malignant transformation of FD lesions later in life, necessitating long-term surveillance. **Table 1** summarizes the age-specific clinical characteristics of MAS (10–17).

**Table 1:** Chronological overview of age-specific clinical manifestations in McCune–Albright syndrome.

Period	Clinical events	Lab tests and clinical considerations	Reported frequency in MAS patients
Infancy (0–1 year)	Café-au-lait macules	Irregular borders (“Maine coastline” pattern), often unilateral along Blaschko lines and not crossing the midline. About 50% are present at birth.	~50%
	Hepatic cholestasis	Jaundice and elevated liver enzymes (ALT/AST) caused by *GNAS* mutations disrupting hepatocyte cAMP signaling and affecting bile excretion.	~20%
	Adrenal cortex hyperfunction	Cushing syndrome, characterized by moon face, hirsutism, and hypertension. Requires cortisol and ACTH testing.	Rare
Early Childhood (1–5 years)	GnRH-independent precocious puberty	Common in female MAS patients, Female-to-male ratio of ~9:1; girls often present with vaginal bleeding and breast development. Ovarian ultrasound may show cysts, rare in male MAS patients.	~85%
	Initial fibrous dysplasia	Affects the femur, tibia, and craniofacial bones, causing bone pain and pathological fractures (“shepherd’s crook” deformity). X-ray/CT shows “ground-glass” changes.	~40%
	Hypophosphatemic rickets	FGF-23–mediated renal phosphate loss causes low serum phosphate and elevated ALP. Manifests as bowlegs, growth retardation, and enamel hypoplasia.	~50%
Childhood (5–12 years)	Scoliosis	FD of the thoracolumbar vertebrae increases the Cobb angle by 5–10° per year. Annual full-spine X-rays are recommended.	~40%
	Craniofacial compression symptoms	Optic nerve compression (15%) and hearing loss (10%). CT shows optic canal narrowing and progressive visual decline.	~25%
	Hyperthyroidism	Tachycardia, sweating, and weight loss. Laboratory tests show suppressed TSH and elevated FT4.	~30%
Adolescence (12–18 years)	Acromegaly	Pituitary Gsa mutation causes elevated GH/IGF-1, often with cranial thickening. Features included enlarged hands and feet and prognathism.	~15%
	Osteosarcoma risk	New bone pain or swelling in long-standing FD lesions may indicate malignant transformation, commonly in the femur or craniofacial bones.	Rare
	Height loss	The primary drivers of height loss are bone age advancement and premature epiphyseal closure caused by precocious puberty, although skeletal factors (such as long bone deformities) may also contribute. Children transformed into central precocious puberty, bone age is advanced by 1.26 ± 0.07 years, with predicted adult height reduced to 152 ± 4.62 cm.	Common
Adulthood (>18 years)	Fixed skeletal deformities	FD progresses slowly, but femoral bowing and spinal kyphosis persist, potentially causing functional impairment.	Common
	Endocrine sequelae	Women: menstrual irregularities, infertility; men: testicular microlithiasis and impaired spermatogenesis.	Common
	Kidney involvement	Fanconi syndrome and kidney failure	Rare
	Malignancy risk	Osteosarcoma and increased risk of breast and thyroid cancers; vigilance is required in patients under 40 years.	Rare

## 3 Histopathological and imaging features

### 3.1 Skeletal lesions

#### 3.1.1 Histopathological features

Intraoperative examination of FD lesions typically reveals expansile masses with relatively well-defined margins. The lesion tissue is often easily separated from the surrounding bone capsule, which is markedly thinned. Grossly, lesions appear gray-brown to gray-yellow and have a firm, gritty texture on sectioning. In some cases, secondary cystic degeneration is present, with accumulation of dark red fluid (18).

**Figure 1:**
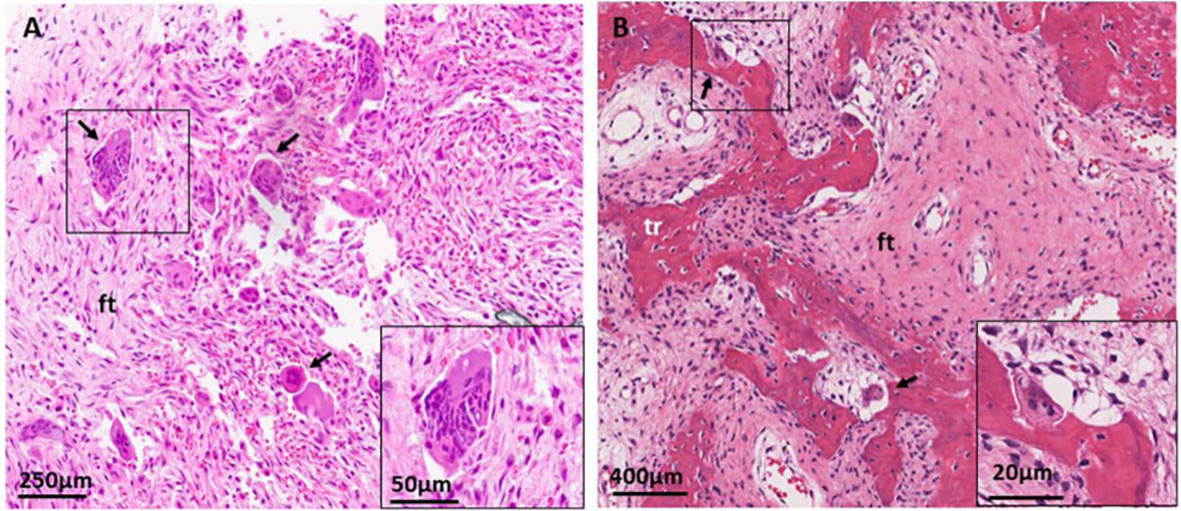
Histological features of craniofacial fibrous dysplasia. **(A)** Hematoxylin and eosin (H&E) staining of the sphenoid bone shows diffuse fibrous tissue (ft) and prominent, irregularly arranged osteoclasts (black arrows). **(B)** Transverse sections of the maxilla demonstrate discontinuously arranged trabeculae within a fibrous tissue, along with osteoclasts (black arrows).

Microscopic evaluation of FD can be influenced by sampling bias. The characteristic histopathological features include a fibrous tissue background with scattered, abnormally formed woven bone, usually arranged in a discontinuous pattern (**Figure 1**) (19). Due to variations in osteoclast abundance and occasional cartilaginous foci, MAS lacks typical bone marrow architecture (20). In craniofacial FD associated with MAS, a pronounced female predominance has been reported, with a male-to-female ratio of approximately 1:9 (21). Affected females may present with vaginal bleeding and premature breast development, often accompanied by ovarian cysts detectable on ultrasonography (13).

#### 3.1.2 Imaging features

The classic radiographic classification of FD (ground-glass, cystic, loofah-like, and moth-eaten patterns) reflects the varying proportions and distribution patterns of fibrous tissue and bone trabeculae within the lesions (22–25). For example, the ground-glass pattern corresponds to fibrous tissue with immature bone trabeculae (22, 23); the cystic pattern corresponds to myxoid degeneration or cystic changes (24); the loofah-like pattern corresponds to mixed fibrous tissue and coarse bone trabeculae (24); and the moth-eaten pattern corresponds to osteolytic areas (25). This imaging-pathology correlation facilitates a better understanding of the nature of FD lesions (see **Table 2**).

**Table 2:** Radiological presentation of fibrous dysplasia.

Pattern	X-ray/CT	MRI	Pathological features
Ground-glass pattern	Expansile thickening of affected bone with cortical thinning; normal bone architecture disappears and is replaced by a homogeneous, trabeculae-free area, producing	T1WI: relatively homogeneous iso- to slightly low signal; T2WI: predominantly heterogeneous iso- to slightly high signal.	Predominantly mature, firm fibrous tissue; reduced fibrous tissue with metaplastic formation of immature, irregularly arranged trabeculae; loose spindle cells in the stroma with abundant mature collagen fibers.
Cystic pattern	Mild irregular expansion and deformation of the diaphysis with cortical thinning and relatively smooth outer margins; small punctate dense foci within cysts. Multicystic lesions show multiple lucent areas of varying size with punctate calcifications and linear bone striations; sclerotic margins	T1WI: low signal; T2WI: markedly high signal with sharp, well-defined borders; internal low-signal linear septations may be present.	Predominantly actively proliferating fibrous tissue with myxoid stromal change and cystic degeneration.
Loofah-like pattern	Coarse, tortuous trabeculae forming thick bone striations oriented along the longitudinal axis.	T1WI: heterogeneous slightly low signal; T2WI: heterogeneous slightly high signal.	Mixed distribution of proliferative fibrous tissue and newly formed trabeculae with variable degrees of maturation.
Moth-eaten pattern	Osteolytic changes with sharp, irregular margins resembling moth-eaten erosion	–	–

### 3.2 Café-au-lait macules of the skin

#### 3.2.1 Histopathological features

Histological examination of café-au-lait macules reveals increased melanin deposition within basal-layer melanocytes and keratinocytes, without melanocyte proliferation (26). In some cases, increased intracellular melanin is also observed within the spinous layer, extending from the granular to the basal layer, in the absence of cytologic atypia. In the dermis, scattered melanophages containing abundant melanin may be present in the superficial layer. Importantly, nerve bundle proliferation and Wagner–Meissner corpuscles are absent, a key feature distinguishing MAS-associated café-au-lait macules from lesions seen in neurofibromatosis.

#### 3.2.2 Light microscopic findings

**Figure 2:**
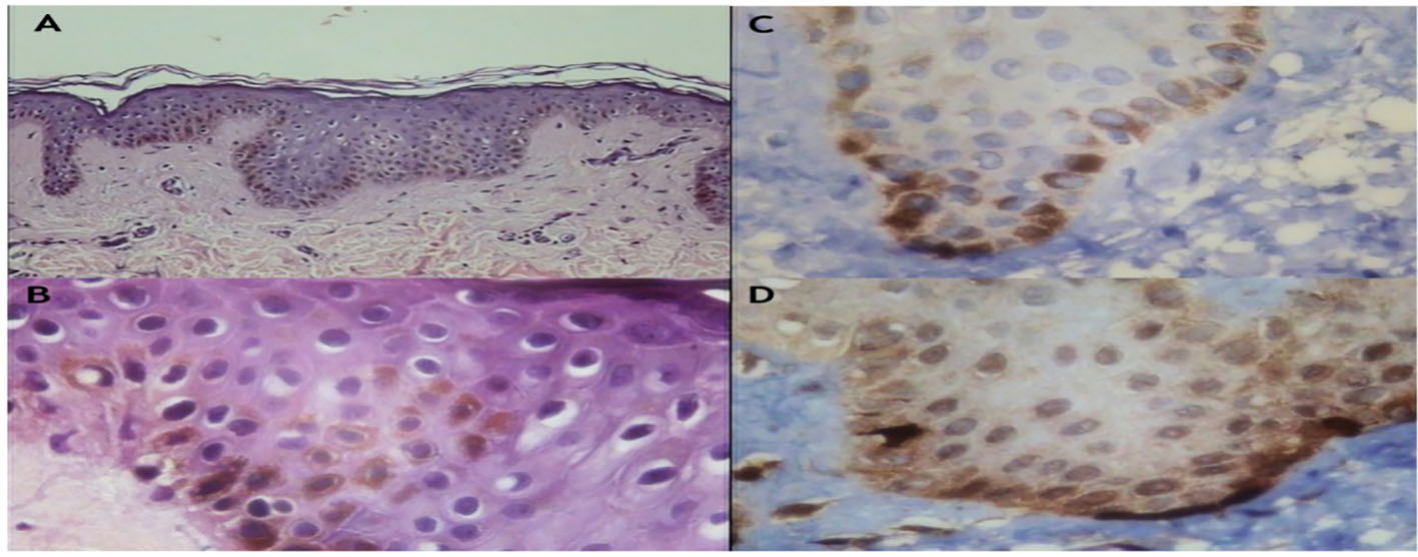
Light microscopy of lesions. **(A)** Increased melanin pigment visualized with H&E staining (×150). **(B)** Enhanced basal-layer pigmentation with scattered melanocytes containing abundant melanin (×400). **(C)** Immunohistochemical staining for Melan-A confirms increased melanin content with a normal melanocyte count (×400). **(D)** Immunofluorescence staining for HMB-45 demonstrates increased melanin content and a normal number of melanocytes (×400).

Light microscopic examination of H&E-stained sections demonstrates increased pigmentation within affected skin regions (**Figure 2a**), with prominent melanin accumulation in the basal layer (**Figure 2b**). Immunohistochemical staining for melanocytic markers HMB-45 and Melan-A shows a normal density of melanocytes (**Figures 2c, d**) (27).

#### 3.2.3 Transmission electron microscopy findings

**Figure 3:**
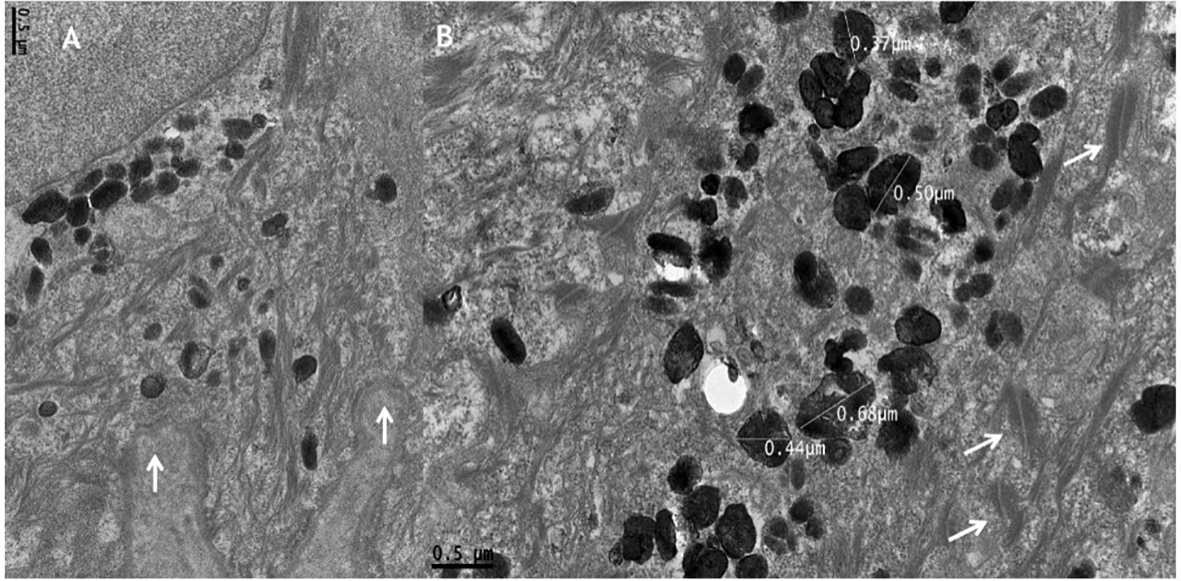
Transmission electron microscopy showing **(A)** a basal keratinocyte with intact hemidesmosomes and normal basement membrane (arrows) (×30,000) and **(B)** numerous heterogeneous melanosomes within keratinocytes, with preserved desmosomes (arrows) (×30,000).

**Figure 4:**
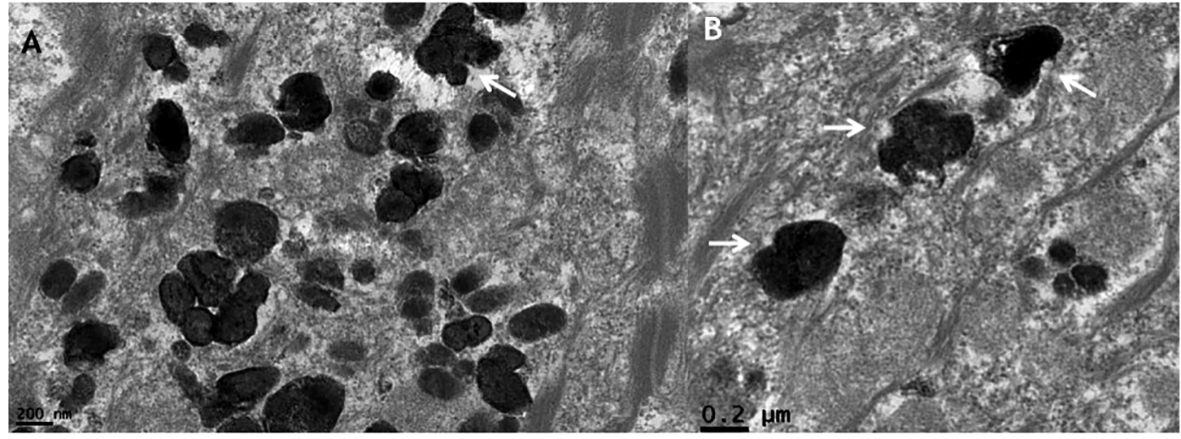
Transmission electron microscopy **(A, B)** showing melanosomes with surface indentations (arrows) (×50,000).

**Figure 5:**
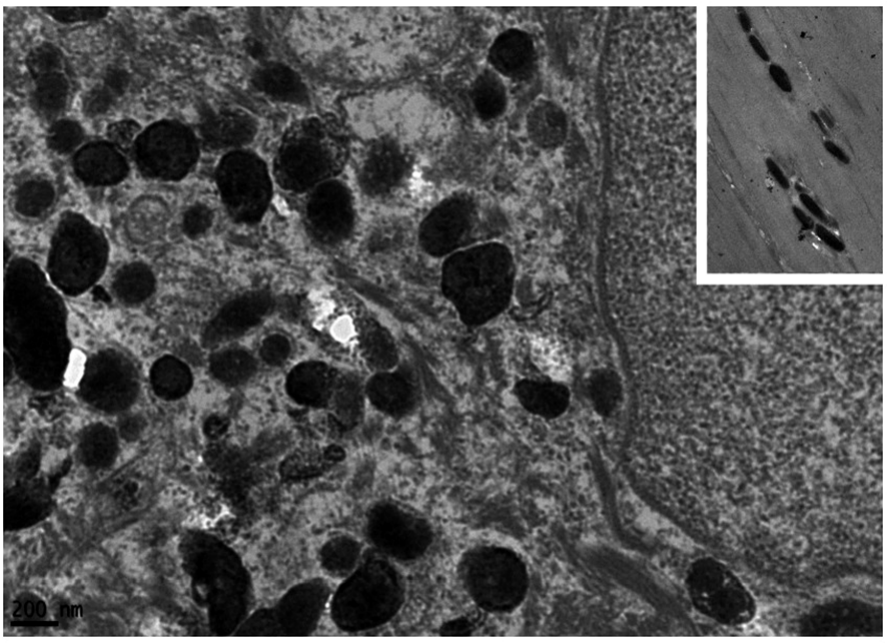
Transmission electron microscopy showing irregular melanosomes, with normal, regular oval melanosomes shown in the inset (×50,000).

TEM analysis reveals preserved epidermal ultrastructure, including intact desmosomes, hemidesmosomes, cytokeratin filaments, and basement membrane (**Figures 3a, b**). Numerous melanosomes are observed within keratinocytes (**Figure 3b**), measuring approximately 0.38–0.67 nm and exhibiting variability in size and morphology (**Figures 3b**, **4a**, **5**). Abnormal melanosomes often display surface indentations (**Figure 4a**) or irregular contours (**Figure 5**) under high magnification, whereas melanosomes in unaffected skin maintain a regular elliptical shape (**Figure 5**) (27).

## 4 Diagnosis and evaluation

### 4.1 Clinical diagnostic criteria

A clinical diagnosis of MAS can be established in patients with FD plus at least one additional major clinical feature, or in the absence of FD, when two or more other major clinical features are present (19, 28). Major features beyond FD include 1) café-au-lait skin pigmentation characteristic of MAS; 2) gonadotropin-releasing hormone (GnRH)-independent precocious puberty and recurrent ovarian cysts in females and GnRH-independent precocious puberty due to autonomous testicular androgen production or ultrasonographic evidence of unilateral/bilateral testicular enlargement with scattered hypoechoic or hyperechoic lesions or microlithiasis in males; 3) thyroid abnormalities consistent with MAS, including bilateral diffuse goiter and multiple high- or low-echo nodules with or without non-autoimmune hyperthyroidism; 4) excess GH secretion; and 5) neonatal ACTH-independent hypercortisolism.

Patients presenting with the complete classical triad can usually be diagnosed following appropriate laboratory, imaging, and endocrine evaluations. However, only approximately 24% of patients with MAS exhibit all three features simultaneously, whereas about 33% present with two features and around 40% manifest only a single feature (29). Therefore, relying solely on the classical triad has limited diagnostic sensitivity and may lead to missed or incorrect diagnoses. In cases with incomplete clinical features, histopathology and molecular genetic testing can provide valuable additional diagnostic support.

## 5 Treatment strategies

MAS is a lifelong disorder. Lesions that develop during childhood frequently persist into adulthood, and affected individuals may experience additional challenges during transitional life stages, including menstrual irregularities, gonadal insufficiency, and fertility impairment. Furthermore, activating *GNAS* mutations are associated with the development of certain malignant tumors. Consequently, long-term surveillance and multidisciplinary management are essential. At present, there is no curative therapy for MAS; therefore, management remains primarily symptomatic and must be individualized according to the heterogeneous clinical manifestations.

### 5.1 Skeletal lesions

To date, no established therapy has been shown to definitively improve bone quality or prevent lesion expansion in FD. Management strategies are directed toward symptom relief, functional optimization, and disability reduction through a combination of physical therapy, pharmacologic treatment, and surgical intervention.

#### 5.1.1 Physical therapy

Low-impact aerobic exercise is recommended for patients with FD, with aquatic therapy regarded as the safest modality for improving muscle strength while minimizing fracture risk. Other low-risk activities include stationary cycling and outdoor bicycling using two- or three-wheeled bicycles (4). These interventions contribute to improved gait mechanics and load distribution, which are critical for preventing progressive deformity, alleviating pain, and reducing late complications such as osteoarthritis. Limb-length discrepancy should be managed conservatively with shoe lifts, while orthoses and assistive walking devices should be individualized based on biomechanics and load-bearing capacity to limit deformity progression. Given the association of FD with cortical thinning, high-impact activities, including jumping and running, as well as resistance training, should be avoided to reduce the risk of pathological fractures.

#### 5.1.2 Pharmacologic therapy: from bisphosphonates to targeted treatments

FD in MAS is a progressive skeletal disorder caused by somatic activating mutations in *GNAS*, resulting in replacement of normal bone with abnormal fibro-osseous tissue. This process leads to reduced bone strength, deformity, and chronic pain. The distinct molecular pathogenesis of FD necessitates pharmacologic approaches that address not only symptom control but also the underlying imbalance in bone remodeling. Therapeutic decision-making is further complicated by the coexistence of self-limited and unpredictably progressive disease courses, substantial heterogeneity in lesion burden and severity, and frequent associations with endocrine hyperfunction, including peripheral precocious puberty, hyperthyroidism, and GH excess. As a result, pharmacotherapy has become a cornerstone of FD management, with three principal objectives: 1) pain relief and improvement of quality of life; 2) suppression of pathological bone resorption to reduce fracture risk; and 3) slowing deformity progression to limit the need for surgical intervention.

The pharmacologic management of FD has evolved from nonspecific analgesic strategies to targeted inhibition of osteoclast-mediated bone resorption. Calcitonin was historically used for bone pain and demonstrated benefit in alleviating pain associated with localized swelling or nerve irritation secondary to fractures. However, due to its inability to improve fibro-osseous bone architecture and high cost, it is now considered a second-line option. Bisphosphonates, owing to their potent antiresorptive properties, are widely used in clinical practice despite the absence of approval by the U.S. Food and Drug Administration (FDA) for MAS/FD. Evidence supporting their use is derived primarily from case reports, case series, and a single randomized controlled trial, with heterogeneous study endpoints (30–33). Pamidronate, a second-generation intravenous bisphosphonate, is commonly administered at a dose of 0.5 mg/kg per infusion (60–90 mg per dose in adults) (12) and has been shown to significantly reduce bone pain, decrease serum alkaline phosphatase and urinary C-terminal telopeptide of type I collagen levels, improve bone strength, and reduce fracture incidence (31). The introduction of third-generation bisphosphonates, including intravenous zoledronate and oral alendronate, has expanded therapeutic options, with recommended doses of 0.075 mg/kg per infusion (4–5 mg per dose in adults) for zoledronate (12) and 40 mg/day for body weight >50 kg, 20 mg/day for 30–50 kg, and 10 mg/day for 20–30 kg for alendronate (30). However, no consensus has been established regarding optimal dosing intervals, which typically vary from every 4–12 weeks to every 6 months based on pain response. Although bisphosphonates are generally effective, disease recurrence after treatment discontinuation is common. While earlier studies reported substantial pain relief and normalization of bone turnover markers, a double-blind randomized controlled trial (30) demonstrated that oral alendronate had no significant effect on pain or functional outcomes, despite its effectiveness in reducing bone resorption and increasing bone density in pediatric patients with FD. In addition, a 2016 study (34) involving 76 patients with FD reported jaw osteonecrosis in 5% of cases, with risk factors similar to those observed in the general population, including prolonged intravenous therapy, high cumulative doses, invasive dental procedures, and poor oral hygiene. Long-term bisphosphonate use may also increase the risk of atypical femoral fractures, as reported in patients treated for osteoporosis, and drug accumulation within bone raises concerns regarding use in women of childbearing potential (35–38). It should be noted that although the efficacy of bisphosphonates in reducing bone pain is widely recognized, the level of evidence for their ability to reduce fracture risk remains low. A systematic review by Regmi et al. (39) involving 92 pediatric patients with FD showed that while all included studies reported significant improvement in bone pain, only some observed a reduction in fracture frequency. Moreover, due to inconsistent definitions of fracture across studies and considerable variability in follow-up duration, a meta-analysis was not feasible. This discrepancy arises because existing studies have largely evaluated fracture as a secondary or exploratory endpoint, lacking prospective randomized controlled trial designs with fracture events as the primary outcome. In the field of FD, the occurrence of fractures is not only related to the level of bone turnover but are also influenced by a combination of factors including skeletal deformities, mechanical axis abnormalities, and local bone fragility at the lesion site. Bisphosphonates cannot correct pre-existing structural defects, which further explains why the evidence base for their role in reducing fracture risk remains relatively weak (39).

Building upon bisphosphonate therapy, denosumab (also known as AMC-162; trade name Prolia), a novel antiresorptive agent targeting the receptor activator of nuclear factor-κB ligand (RANKL) pathway, has shown potential benefit in patients with refractory skeletal disease, intolerance or inadequate response to conventional therapies, or lesions unsuitable for surgical management. Denosumab specifically blocks RANKL, an osteoclast-activating factor that is abnormally upregulated in FD lesions, thereby markedly suppressing pathological bone turnover and improving bone architecture and pain. On May 28, 2010, denosumab was approved by the European Commission for the treatment of severe osteoporosis in postmenopausal women. In comparable populations, denosumab achieved earlier pain control by 2 weeks than alendronate and further increased bone mineral density, without significant differences in adverse events such as elevated serum N-terminal telopeptide of type I collagen or hypocalcemia (40). In reported MAS/FD cases, denosumab has been used as a last-line, off-label therapy, most commonly administered subcutaneously at doses of 60 mg every 3–6 months or 120 mg every 6 months, often with an initial loading regimen during the first 4 weeks of treatment (41). Available evidence suggests that denosumab is effective in metabolically active FD lesions (42–44). However, unlike bisphosphonates, its antiresorptive effect is short-lived, and treatment discontinuation may result in hypophosphatemia, rebound hypercalcemia, accelerated bone loss, and increased fracture risk, thereby limiting its widespread clinical application (45, 46). In addition, hypocalcemia may occur during the early phase of treatment (47). Therefore, careful assessment of risks and benefits is essential before initiating denosumab therapy.

#### 5.1.3 Surgical intervention strategies for different anatomical sites

Surgical intervention plays a critical role in the management of FD by correcting skeletal deformities, relieving neural compression, and restoring function, particularly in patients with medically refractory disease or severe complications such as pathological fractures, threatened vision, or significant functional impairment. Surgical decision-making should be individualized, taking into account lesion location, progression rate, and age-related considerations.

##### 5.1.3.1 Craniofacial FD

Surgical management of craniofacial FD, particularly when the skull base is involved, is especially challenging in pediatric patients because of complex regional anatomy and proximity to critical neurovascular structures. In such cases, the potential benefits of intervention must be carefully balanced against operative risks. Surgery should generally be avoided during periods of active disease, as it neither cures FD nor reliably halts disease progression. Indications for intervention include severe proptosis, diplopia, optic nerve compression, and cranial nerve palsies (48).

##### 5.1.3.2 Optic nerve decompression

The management of optic nerve compression in FD remains controversial. In patients with absent or mild compression, close monitoring is recommended, with particular attention to coexisting GH excess. In cases of acute visual deterioration or vision loss, prompt magnetic resonance imaging should be performed to exclude secondary lesions such as aneurysmal bone cysts or mucous cysts. When surgical intervention is indicated, decompression should ideally be performed within one week or sooner (48). Some investigators have suggested that acute visual loss may result from hemorrhage due to rupture of an aneurysmal bone cyst or from rapid lesion expansion causing optic nerve compression and have advocated adjunctive medical management with acetazolamide to reduce intracanal pressure (48).

Primary optic nerve decompression in FD may be performed using either traditional open surgical techniques or endoscopic approaches. Open surgery provides wide exposure and direct visualization of the superior and lateral walls of the optic canal but is highly invasive and associated with substantial intraoperative bleeding, brain retraction-related injury, cerebrospinal fluid leakage, intracranial infection, and permanent facial scarring. Consequently, endoscopic approaches have become preferred in many centers. Endoscopic decompression via a burr-hole corridor offers minimal invasiveness, reduced bleeding, shorter operative time, and faster postoperative recovery. However, this technique is technically demanding, particularly in cases of extensive skull-base hyperostosis, and outcomes are highly dependent on surgical expertise. In addition, the approach is generally not feasible in children younger than 2 years because narrow piriform apertures limit instrument access. In children aged 2 years or older, anatomical dimensions are usually sufficient to accommodate the endoscope and necessary instruments. Multiple studies have demonstrated the safety and efficacy of endoscopic approaches in pediatric patients, with acceptable morbidity profiles (49–53). An additional challenge in pediatric endoscopic skull-base surgery is incomplete sphenoid sinus pneumatization, which necessitates extensive drilling to access the sella, optic nerve, and sphenoid planum. Intraoperative navigation systems partially mitigate the lack of anatomical landmarks, enabling more precise identification of surgical boundaries and critical structures (50). Given the unpredictable and progressive nature of FD, recurrence rates and the need for reoperation remain high. A multicenter retrospective study by Fang et al. (54) revealed that the postoperative clinical recurrence rate in patients with craniofacial FD was 38.89%. Further analysis identified younger age at disease onset and at surgery, elevated preoperative serum alkaline phosphatase (ALP) levels, debulking surgery, and polyostotic lesions as significant contributing factors to the higher clinical recurrence rate. Univariate and multivariate analyses further confirmed that polyostotic lesions as an independent risk factor for postoperative clinical recurrence. Additionally, the study suggested that elevated preoperative ALP levels could serve as a valuable consideration in surgical planning. A study by Chen et al. (55) involving 93 surgically treated patients with craniofacial FD/MAS, with a follow-up period of 20 to 40 years (mean 30 years), provided important data for understanding postoperative outcomes. The results showed that among 10 patients who underwent radical resection and immediate bone graft reconstruction for Zone 1 (fronto-orbital, zygomatic, and maxillary regions) lesions, 2 experienced recurrences, suggesting that this strategy can achieve favorable long-term control. Among 3 patients who underwent therapeutic optic nerve decompression for Zone 3 (central cranial base) lesions, 2 had postoperative visual improvement, supporting the principle of considering surgical intervention only in cases of progressive visual decline. The study concluded that surgical strategies based on the four-zone classification yield satisfactory outcomes: for Zone 1 lesions, radical resection followed by immediate bone graft reconstruction is feasible; for Zone 3, therapeutic rather than prophylactic optic nerve decompression is indicated. Accordingly, most reports advocate conservative management in children, reserving surgical intervention for cases in which neural injury is likely to be alleviated or reversed by decompression. Prophylactic optic nerve decompression is generally considered contraindicated.

##### 5.1.3.3 Proximal femoral FD

The incidence of fractures in FD varies with age and peaks between 6 and 10 years, during which affected individuals experience an estimated average of 0.4 fractures per year (56). The femur is the most frequently involved site in polyostotic FD, particularly the proximal third, often presenting with coxa vara and the characteristic “shepherd’s crook” deformity. FD-affected bone is structurally fragile, highly vascularized, and frequently characterized by obliteration of the medullary canal, rendering surgical correction particularly challenging. Curettage and bone grafting using autologous or allogeneic grafts have been widely employed but are often ineffective in polyostotic disease, as grafted bone is typically resorbed over time (57, 58). Corrective osteotomy combined with intramedullary fixation, particularly using interlocking nails, is currently considered the preferred surgical strategy for polyostotic FD, whereas plate-and-screw constructs are not recommended, except in selected cases involving stabilization of isolated simple coxa vara (59–61). A retrospective study by Jyoti et al. (62) involving eight patients with a clinical diagnosis of proximal femoral FD proposed a novel combined treatment strategy, namely zoledronic acid therapy combined with valgus osteotomy. Postoperative follow-up at one year showed that pain symptoms were relieved in all eight patients, with complete healing and remodeling at the osteotomy site, complete resolution of pain, and only a mild limp. A case report by Zhao et al. (63) described the use of the bridging combination internal fixation system for the treatment of a pathological femoral fracture caused by FD, offering a new option for the surgical management of proximal femoral FD. The patient was a 29-year-old male diagnosed with polyostotic FD, presenting with a shepherd’s crook deformity and a pathological fracture in the subtrochanteric region. The researchers applied the bridging combination internal fixation system for the first time. Postoperative fracture healing was assessed using the RUSH score, which was 9, 22, and 28 at 1, 3, and 6 months, respectively. The left hip function score improved from 28 preoperatively to 88 at 6 months postoperatively, indicating satisfactory fracture healing and good recovery of hip function. This system offers advantages such as minimally invasive application, stable fixation, and easy contouring, providing an effective solution for treating pathological femoral fractures caused by FD.

##### 5.1.3.4 Tibiofibular FD

As important weight-bearing structures of the lower limb, the tibia and fibula can also sustain pathological fractures in patients with MAS, and their management presents significant challenges. Zhu et al. (64) reported a case of a tibiofibular fracture in a patient with MAS treated with cross-union surgery. The patient was a 14-year-old girl who sustained a distal tibiofibular fracture following trauma. After conservative treatment, she developed nonunion, characterized by an inability to bear weight and external rotation of the lower leg. Given the propensity for nonunion after fractures in children with MAS and the high failure rate of conventional treatments, the researchers applied the “cross-union” technique, originally used for congenital pseudarthrosis of the tibia, to this patient. During the procedure, a Fassier-Duval rod combined with a locking plate was used for tibial fixation, and K-wires were used for fibular fixation. Autologous iliac crest bone and a periosteal graft were harvested and transplanted, combined with bone morphogenetic protein-2 to promote healing. At the 12-month postoperative follow-up, a stable bone union at the distal tibia and fibula was observed, with no refracture. The Radiographic Union Score for Tibial fractures was 12, indicating complete healing, and the Olerud Molander Ankle Score was 60, reflecting improvement in pain and swelling but with mild limitations in activity. This technique may offer an effective salvage option for complex fractures in MAS; however, it remains in the preliminary stages of application, and its long-term efficacy requires validation through more cases.

### 5.2 Endocrine hyperfunction

#### 5.2.1 Management of female peripheral precocious puberty

Current pharmacological management of female PPP primarily involves aromatase inhibitors (AIs) and estrogen receptor antagonists, which are often used sequentially or in combination in clinical practice. AIs suppress the conversion of androgens to estrogens, thereby reducing local estrogen levels around bone tissue, delaying epiphyseal closure, and improving final adult height outcomes. Third-generation AIs, owing to their enhanced efficacy and more favorable safety profiles, have become the cornerstone of PPP management in females. Letrozole, a representative agent, binds specifically to the heme group of aromatases, with an inhibitory potency more than 1,000-fold greater than that of first-generation agents. The recommended dosage is 1–2 mg/(m²·day), with a maximum daily dose of 2.5 mg, administered orally once daily for a minimum of 6 months. Treatment duration should be individualized based on PPP recurrence. In a single-center retrospective study involving 28 girls treated with letrozole, bone age advancement (ΔBA/ΔCA) declined significantly from a median of 1.7 to 0.5, growth velocity Z-scores decreased from 2.2 ± 2.3 to 0.6 ± 1.6 and predicted adult height Z-scores improved significantly from 2.9 ± 3.2 to 0.8 ± 1.5. Throughout treatment, uterine and ovarian volumes remained stable, and no treatment-related adverse events were reported. Notably, four patients achieved normal adult height, a result that reached statistical significance despite the limited sample size (65). A subset of patients may exhibit estrogen “escape” during letrozole therapy, characterized by rising circulating estrogen levels despite ongoing treatment. In such cases, combination therapy or transition to second-line agents, including tamoxifen or fulvestrant, may be considered. Clinical trials have demonstrated that tamoxifen significantly reduces the frequency of vaginal bleeding; however, its use is associated with estrogen withdrawal bleeding, an increased risk of endometrial pathology, and androgenic adverse effects such as hirsutism, which occur in approximately 20% of patients. Consequently, tamoxifen should be used with caution. The recommended dosage is 0.4-0.6 mg/(kg·day), administered in divided doses, with a maximum daily dose of 30 mg. Fulvestrant, a competitive estrogen receptor antagonist, differs fundamentally from both aromatase inhibitors and selective estrogen receptor modulators. It functions as a pure estrogen antagonist by irreversibly binding to estrogen receptors, inducing conformational changes that promote ubiquitin-mediated receptor degradation and thereby completely abrogating estrogen signaling. Importantly, fulvestrant lacks partial agonist activity, eliminating the risk of endometrial hyperplasia observed with tamoxifen therapy. In an international multicenter prospective study (66), 30 females aged ≤10 years with MAS-associated progressive PPP received intramuscular fulvestrant at a dose of 4 mg/kg monthly for 12 months. Treatment led to reductions in the duration and frequency of vaginal bleeding, deceleration of bone age progression, stabilization of uterine volume, and no serious adverse events. Nevertheless, fulvestrant is currently approved primarily for the treatment of tamoxifen-resistant, estrogen receptor–positive breast cancer. Its use in MAS remains limited, and to date, no clinical experience has been reported in China.

#### 5.2.2 Management of male PPP

In boys with MAS, PPP results from constitutive activation of the Gsα protein, which stimulates Leydig cells via the cAMP–PKA signaling pathway, leading to autonomous testosterone secretion. Excess testosterone not only directly drives the premature onset of masculinizing features but also undergoes peripheral aromatization to estrogen, accelerating epiphyseal closure and resulting in advanced bone age, often exceeding chronological age by 2 years or more, with severe compromise of predicted adult height. Accordingly, pharmacological management targets two principal mechanisms: suppression of androgen action and inhibition of estrogen production. Antiandrogen therapy aims to block androgen receptors or inhibit testosterone synthesis and includes agents such as spironolactone and ketoconazole. Aromatase inhibition, typically achieved with letrozole and anastrozole, prevents the conversion of testosterone to estrogen and is a critical component of therapy. Spironolactone, an aldosterone antagonist with antiandrogenic properties, competitively inhibits dihydrotestosterone binding to androgen receptors. This results in reductions in serum free testosterone levels, acne, pubic hair development, and aggressive behavior. However, spironolactone alone does not adequately suppress bone age progression and should therefore be combined with an aromatase inhibitor. Ketoconazole inhibits cytochrome P450 enzyme CYP17, thereby suppressing testosterone synthesis. Its use is generally reserved for patients with extremely elevated testosterone levels or resistance to GnRH agonists, but its clinical utility is limited by a substantial risk of hepatotoxicity. Tessaris et al. (67)reported a case of a boy with molecularly confirmed MAS who developed rapidly progressive peripheral precocious puberty at 4.6 years of age. He was treated with a combination of bicalutamide (25 mg/day) and anastrozole (1 mg/day) for 49 months. The results showed rapid normalization of growth velocity, reduced penile androgenization, stable testicular volume, and no observed adverse effects or secondary central precocious puberty activation. This case provides important evidence for the efficacy and safety of combined antiandrogen and aromatase inhibitor therapy in the management of PPP in boys with MAS.

#### 5.2.3 Gonadotropin-independent precocious puberty transform into gonadotropin-dependent precocious puberty

During the management of MAS, a noteworthy clinical phenomenon is the dynamic evolution of precocious puberty types. Due to autonomous ovarian hyperfunction, patients with MAS are chronically exposed to elevated estrogen levels. This prolonged exposure can lead to premature activation of the hypothalamic-pituitary-ovarian axis (HPOA), causing the initial GIPP to transform into secondary GDPP. A typical case reported by Agarwal et al. (68)clearly illustrates this transformation and its clinical management. The patient was a 7-year-old girl who presented with unilateral breast development. A physical examination revealed café-au-lait macules on her back, leading to a clinical diagnosis of MAS. At the initial diagnosis, a GnRH stimulation test confirmed GIPP, consistent with the characteristic pathophysiology of MAS-autonomous ovarian estrogen secretion independent of central GnRH drive. During the GIPP phase, the patient was treated with letrozole. By inhibiting the conversion of androgens to estrogen, letrozole effectively reduces peripheral estrogen levels, thereby controlling the development of secondary sexual characteristics, slowing bone age advancement, and reducing episodes of vaginal bleeding. After one year of treatment, a repeat GnRH stimulation test indicated a conversion to GDPP, suggesting that prolonged exposure to high estrogen levels had induced premature activation of the HPOA. Following this transformation, the treatment strategy was adjusted accordingly. Therefore, in addition to continuing to control peripheral estrogen production, therapy targeting central precocious puberty was introduced. Consequently, the patient was started on a GnRH agonist-leuprolide (3.75 mg intramuscularly once monthly). Leuprolide acts by continuously stimulating pituitary GnRH receptors, leading to receptor desensitization and downregulation. This suppresses the secretion of gonadotropins (LH and FSH), thereby halting centrally-driven pubertal progression.

#### 5.2.4 Hyperthyroidism

Hyperthyroidism is the second most common endocrine abnormality in patients with MAS and occurs at a markedly higher prevalence than in the general population. Methimazole is the preferred first-line pharmacological agent and acts by inhibiting thyroid peroxidase, thereby reducing iodine organification and thyroid hormone synthesis. Dosing generally follows standard pediatric hyperthyroidism protocols. A study by Tessaris et al. (69) involving 36 children with MAS identified hyperthyroidism in 4 cases, presenting as either diffuse (2 cases) and nodular (2 cases) goiter. These patients demonstrated favorable clinical and biochemical responses following treatment with methimazole (0.2-0.5 mg/kg/day). However, in contrast to Graves’ disease, MAS-associated hyperthyroidism demonstrates a poor response to antithyroid drugs (ATDs). Feuillan et al. (70) reported that only one of 19 MAS patients with thyrotoxicosis achieved long-term remission with medical therapy. This limited responsiveness is attributed to the persistent presence of activating *GNAS* mutations, which confer autonomous thyroid hormone production that is not adequately suppressed by ATDs. For patients with hyperthyroidism persisting beyond 5 years, definitive treatment with thyroidectomy or radioactive ablation is recommended. Radioiodine (I^131^) therapy may be considered; however, careful pre-treatment evaluation of thyroid nodules is essential. Given the potential for thyroid tissue regrowth, long-term annual surveillance is advised following definitive therapy. Thyroidectomy should be favored in patients with clinically significant nodules, compressive symptoms, or poor control of thyrotoxicosis. Because radiotherapy may increase the risk of thyroid carcinoma, its routine use is generally discouraged.

#### 5.2.5 GH excess

GH excess in MAS arises from somatic *GNAS* mutations that lead to autonomous activation of pituitary somatotrophs. Persistent stimulation of the cAMP–PKA pathway leads to excessive GH secretion and elevated insulin-like growth factor 1 (IGF-1) levels. A multicenter cross-sectional analysis by Tessaris et al. (71) compared 37 MAS patients with GH excess to 34 MAS controls without GH hypersecretion. The study found that the mean head circumference standard deviation score was significantly higher in the GH excess group, suggesting more severe craniofacial skeletal involvement. Regarding complication risks, GH excess significantly increased the odds ratios for optic neuropathy, hearing impairment, and facial asymmetry, among other FD-related comorbidities, revealing the profound impact of GH-IGF-1 axis hyperactivity on fibrous dysplasia (FD) lesions. More critically, the study demonstrated that the risk of malignancies in the GH excess group was 15.24 times higher than in the control group. This research indicates that GH hypersecretion in MAS is associated with a significantly increased burden of both comorbidities and oncological risk. In pediatric patients, the primary therapeutic goal is normalization of IGF-1 to age- and sex-adjusted population medians, whereas in adults, maximal feasible reduction of IGF-1 levels is pursued. Management strategies include medical therapy, surgery, and radiotherapy, with medical therapy regarded as first-line treatment. Long-acting somatostatin analogs, such as octreotide, are commonly used as initial therapy. Second-line treatment consists of the GH receptor antagonist pegvisomant, which may be administered alone or in combination with octreotide or lanreotide. The study by Tessaris et al. (71) demonstrated that pharmacological treatment (octreotide monotherapy at 10–30 mg/month, or in combination with pegvisomant at 10–20 mg/day) effectively achieved normalization of IGF-1 in 72.4% (21/29) of compliant patients. If pharmacotherapy fails, total hypophysectomy may be indicated as the disease typically involves the entire gland. Adenoma resection alone is insufficient to control GH excess. Image-guided transsphenoidal surgery has been shown to be an effective and relatively safe option in selected patients. Radiotherapy is reserved for cases refractory to both medical and surgical treatment, given the increased risk of malignant transformation within FD lesions.

#### 5.2.6 Cortisol excess

Cortisol excess in MAS frequently originates in fetal life, exposing the developing fetus to elevated glucocorticoid levels. In up to one-third of cases, hypercortisolism resolves spontaneously after birth, and mild cases may therefore be managed conservatively with close observation (12). In severe or persistent disease, bilateral total adrenalectomy is typically required, resulting in permanent adrenal insufficiency and the need for lifelong hormone replacement. In stable patients with unilateral adrenal involvement, unilateral adrenalectomy may be considered as an alternative. For patients who are not suitable surgical candidates, medical therapy with agents such as metyrapone, ketoconazole, or mitotane may be employed. Ketoconazole, however, must be administered cautiously due to its hepatotoxicity.

#### 5.2.7 MAS-associated FGF23 hypophosphatemia

Traditional treatment for MAS-related hypophosphatemia has primarily followed the regimen established for X-linked hypophosphatemia (XLH), namely oral phosphate supplements combined with active vitamin D (calcitriol or alfacalcidol). However, this approach has limited efficacy in patients with MAS and carries risks of hypercalciuria, hypercalcemia, secondary hyperparathyroidism, and gastrointestinal adverse effects, requiring particular caution in those with pre-existing nephrocalcinosis (72). The advent of burosumab has provided a novel therapeutic option for MAS-associated FGF23-mediated hypophosphatemia. Stelmachowska-Banaś et al. (73) first reported the use of burosumab in an adult patient with MAS. This 27-year-old man presented with multiple fractures, bone pain, and muscle weakness that remained uncontrolled despite treatment with oral phosphate and calcitriol. Following initiation of burosumab at 1 mg/kg administered subcutaneously every 4 weeks, parameters of calcium-phosphate metabolism normalized, and both alkaline phosphatase activity and its bone-specific fraction decreased substantially. No new fractures occurred during 24 months of treatment, and the patient reported reduced bone pain and improved quality of life, with no adverse events observed. The most representative pediatric case was reported by Saito et al. (74) in a 2-year-old girl, representing the youngest patient treated with burosumab for MAS. In addition to FD and FGF23-related hypophosphatemia, this patient had a particularly severe phenotype including hyperthyroidism, Cushing syndrome, GH excess, and gonadotropin-independent precocious puberty. She had sustained more than seven fractures before 16 months of age. Burosumab (1.0 mg/kg every 2 weeks) was initiated at 2 years and 3 months of age. Within one month, serum phosphate levels increased from 3.2 mg/dL to 5.8 mg/dL, and the ratio of tubular maximum reabsorption of phosphate to glomerular filtration rate (TmP/GFR) rose from 2.49 mg/dL to 4.59 mg/dL. Following her last femoral diaphyseal fracture at 2 years and 7 months of age, no further fractures occurred through the most recent follow-up at 4 years and 6 months of age. Bone pain significantly diminished, and the patient, who had been unable to stand, recovered to the point of being able to stand with support. However, radiographic evaluation during burosumab treatment revealed no improvement in the patient’s FD lesions.

As a monoclonal antibody targeting FGF23, burosumab has demonstrated rapid and sustained biochemical improvements and significant clinical benefits in both pediatric and adult patients. Although it has not yet been shown to reverse radiographic progression of FD lesions, burosumab represents a breakthrough in the treatment of MAS-related hypophosphatemia and holds promise as a future standard of care for MAS-associated FGF23-mediated hypophosphatemia.

### 5.3 Café-au-lait macules treatment

Café-au-lait macules associated with MAS do not undergo malignant transformation and generally require no treatment unless cosmetic concerns arise (75). Until recently, no effective pharmacological therapies were available for these lesions. Laser therapy remains the primary treatment modality, with commonly used systems including Q-switched Nd: YAG, Q-switched alexandrite, Q-switched ruby, copper vapor, and pigment-specific dye lasers. Multiple treatment sessions are typically required to achieve satisfactory lesion clearance. Potential risks include hypopigmentation, transient or permanent hyperpigmentation, scarring, and incomplete lesion resolution.

### 5.4 Transition management

As a lifelong condition, MAS requires careful transition planning from late adolescence, generally from 16 years of age onward, into adult care. Transition management should address ongoing endocrine disorders, persistent bone metabolism abnormalities, and fertility preservation. Pediatric care providers should proactively educate patients and families, perform structured evaluations, and coordinate referrals to appropriate adult subspecialists to ensure continuity of care and support lifelong follow-up.

## 6 Outcomes and prognosis

MAS prognosis is highly variable and is influenced by the degree of *GNAS* mutation mosaicism, the specific organs involved, and the timing of clinical intervention. Early and systematic management can significantly improve quality of life; however, persistent challenges remain, including progressive skeletal deformities, an increased risk of malignant transformation, and long-term endocrine sequelae. Mortality in MAS is most commonly attributable to cardiopulmonary complications, with significantly increased mortality in patients with concomitant progressive gigantism, heart failure, or recurrent pathological fractures. For example, one patient with MAS complicated by a GH-secreting pituitary adenoma treated at Peking Union Medical College Hospital died as a result of cardiac disease (76). Approximately 30% of adult patients experience chronic bone pain, gait impairment, or pathological fractures related to FD, with an estimated annual fracture incidence of approximately 8%, often necessitating the use of assistive devices or wheelchairs (16). Additionally, psychosocial burden is substantial; nearly 60% of adolescents report body image disturbances related to pigmentary lesions or skeletal deformities, and the risk of depression in this population is estimated to be twice that observed in individuals with other chronic diseases (77). A prospective observational cohort study by Bulaicon et al. (78) involving Dutch patients diagnosed with FD/MAS demonstrated that, over time, these individuals may experience a range of issues including emotional distress, social difficulties, sleep problems, and lower life satisfaction. Research by Legrand et al. (79) indicated that neuropathic pain is more pronounced in female patients, and that generalized pain is significantly associated with sleep impairment, depression, and moderate to severe anxiety.

These findings underscore that the management of MAS should not be confined to the control of physical symptoms but must also incorporate mental health assessment and intervention. Long-term management of MAS requires attention to both its physical and psychological dimensions to optimize overall patient outcomes.

## 7 Challenges and perspectives

In summary, despite significant advances in elucidating the etiology, genetics, diagnostic approaches, and treatment strategies for MAS, the underlying genetic basis remains unidentified in a subset of patients, suggesting potential pathogenic genes or epigenetic mechanisms. Future research should prioritize the identification of these factors to fully elucidate the molecular pathology of MAS. The mosaic distribution of activating *GNAS* mutations (e.g., R201H/C), leads to pronounced tissue-specific phenotypic variability, and currently available pharmacological therapies are unable to fully suppress the aberrant cyclic AMP–protein kinase A signaling pathway. Therefore, developing more precise targeted therapies represents a critical unmet need. As MAS/FD are rare diseases, significant gaps remain in our understanding of their natural history and clinical outcomes. Heterogeneity of data across different medical centers, a lack of uniformity in follow-up protocols, and inconsistency of outcome measures all limit our comprehensive grasp of disease patterns and objective evaluation of treatment efficacy. Establishing multicenter standardized datasets is crucial for improving disease knowledge and management of FD/MAS. This multicenter collaborative data-sharing model can not only accelerate the progress of clinical research but also facilitate the formation development of evidence-based diagnostic and therapeutic consensus. Although early diagnosis and timely intervention can improve functional outcomes and quality of life, many patients continue to experience recurrent fractures, an ongoing risk of malignant transformation, and persistent endocrine complications. Establishing multidisciplinary, long-term follow-up frameworks that integrate endocrine surveillance, skeletal monitoring, and assessment of associated systemic complications is crucial for optimizing long-term outcomes in patients with MAS.
